# Which vaginal douching agent is the best choice before oocyte retrieval? A systematic review and network meta-analysis

**DOI:** 10.3389/frph.2022.1032062

**Published:** 2022-11-04

**Authors:** Ying Meng, Gui H. Wen, Hong Luo, Xiu C. Tan, Li Wang, Juan Liao, Hong Peng, Ling Lan, Na Yang, Ying Zhao

**Affiliations:** Center for Reproductive Medicine, Women and Children's Hospital of Chongqing Medical University, Chongqing, China

**Keywords:** vaginal douching, oocyte retrieval, pelvic infection, IVF outcome, network meta analysis

## Abstract

**Objective:**

The aim of this study was to evaluate six vaginal douching agents (Iodine, Saline, Iodine followed by saline, chlorhexidine acetate followed by saline, Ozone, Potassium permanganate) on oocytes pick-up related pelvic infection (OPU-PI) and IVF outcome in patients underwent assisted reproduction technology (ART).

**Design:**

Through searching PubMed, Embase, Cochrane Library, Web of Science, Ovid, CINAHL CNKI, only human clinical trials were collected to study the effects of the six vaginal douching agents on OPU-PI and IVF outcomes. The included studies were evaluated for methodological quality by the Cochrane bias risk assessment tool, and the data analysis software was used to analyze the data accordingly.

**Results:**

The clinical trials were collected between the earliest available date and June 2022. Eight studies were included, the total sample size used in the study was 12,567. The results of the network meta-analysis showed that Ozone can significantly decrease OPU-PI; Iodine followed by saline can be a antiseptic protocol ranked first without affecting the quality of oocytes and Chlorhexidine acetate followed by saline can improve patients' clinical pregnancy rate.

**Conclusion:**

Based on Ranking Plot of the Network, this review reports the best evidence available regarding different vaginal douching agents used before OPU.

## Introduction

Infertility is common, with recent publications quoting a 9% to 18% prevalence in the general population (1). Approximately 12.7% of reproductive age women seek treatment for infertility each year (2), many of whom will ultimately require assisted reproductive technology (ART). Ultrasound-guided oocyte retrieval is an essential part of ART. Even though oocyte retrieval is seen as a safe and efficient procedure, it is considered a process that potentially occur surgery- or infection-related complications (3). The incidence of oocyte pick-up related pelvic infection (OPU-PI) has been reported as 0.02% by the European Society of Human Reproduction and Embryology data, other studies report an incidence rate of up to 0.3–1.5% (4, 5). It is widely agreed that pre-operative vaginal preparation cannot rule out the infection in all cases because every imaginable effort is made to minimize the risk of pelvic infections (6). However, the use of antiseptic solutions on vaginal douching before OPU in ART is controversial. Two major concerns regarding the use of antiseptic solutions in patients actively undergoing OPU with *in vitro* fertilization (IVF) are benefits for reducing complications related to OPU and the toxicity impact on quality of oocytes (7, 8).

A recent study showed that preoperative vaginal douching with distilled water led to a significantly higher rate of postoperative infection than did preoperative vaginal irrigation with Iodine (9). However, some douching agents were confirmed associated with epithelial disruption and inflammation, and may reduce the anti-inflammatory effects of beneficial lactobacilli (10). It is therefore particularly important to find a vaginal douching product used before OPU with effectiveness against infections and whether it affects IVF outcome should be considered.

There is no uniform standard of vaginal preparation before OPU, different vaginal douching agents were used in different countries/regions. Network meta-analysis is a technique that uses direct or indirect comparisons to compare the effects of multiple interventions on a disease and to estimate the rank order of each treatment (11). Therefore, in this study we used network meta-analysis to compare different vaginal preparation (Iodine, saline, Iodine followed by saline, chlorhexidine acetate followed by saline, potassium permanganate and ozone) in order to assess the effect of these agents on OPU-PI and IVF outcome and to provide patients and clinicians with a better advice about the effects of these vaginal irrigation agents. The aim is to evaluate the effects of these vaginal douching agents on OPU-PI and IVF outcome and to provide evidence-based recommendations for patients and clinicians.

## Methods

This systematic review and network meta-analyses are reported according to the PRISMA statement guidelines. The review was registered with the PROSPERO database (Registration number: CRD42022306779).

### Search strategy

The researchers searched seven electronic databases (Pubmed, EMBASE, Cochrane Central Register of Controlled Trials, Web of Science, CINAHL, Ovid and CNKI) from their creation to 30 June 2022. The search strategy was constructed around the PICOS tool: (P) Population: patients need vaginal prep before oocyte retrieval in IVF-ET; (I) Intervention: using Iodine to wash the vagina before oocyte retrieval; (C) Comparator: using other vaginal irrigation agents to wash the vagina; (O) Outcomes: OPU-PI and IVF outcome (including fertilization rate and clinical pregnancy rate). (S) Study type: human clinical trials. The detailed search strategy is shown in Table 1 (Pubmed is used as an example).

**Table 1:** Search strategy on pubmed.

#1	“oocyte retrieval"[MeSH Terms] OR (“oocyte"[All Fields] AND “retrieval"[All Fields]) OR “oocyte retrieval"[All Fields] OR (“oocyte retrieval"[MeSH Terms] OR (“oocyte"[All Fields] AND “retrieval"[All Fields]) OR “oocyte retrieval"[All Fields] OR (“retrieval"[All Fields] AND “oocyte"[All Fields]) OR “retrieval oocyte"[All Fields]) OR (“oocyte retrieval"[MeSH Terms] OR (“oocyte"[All Fields] AND “retrieval"[All Fields]) OR “oocyte retrieval"[All Fields] OR (“oocyte"[All Fields] AND “collection"[All Fields]) OR “oocyte collection"[All Fields]) OR (“oocyte retrieval"[MeSH Terms] OR (“oocyte"[All Fields] AND “retrieval"[All Fields]) OR “oocyte retrieval"[All Fields] OR (“collection"[All Fields] AND “oocyte"[All Fields]) OR “collection oocyte"[All Fields]) OR (“oocyte retrieval"[MeSH Terms] OR (“oocyte"[All Fields] AND “retrieval"[All Fields]) OR “oocyte retrieval"[All Fields] OR (“oocyte"[All Fields] AND “aspiration"[All Fields]) OR “oocyte aspiration"[All Fields]) OR (“oocyte retrieval"[MeSH Terms] OR (“oocyte"[All Fields] AND “retrieval"[All Fields]) OR “oocyte retrieval"[All Fields] OR (“aspiration"[All Fields] AND “oocyte"[All Fields]) OR “aspiration oocyte"[All Fields]) OR ((“oocyte s"[All Fields] OR “oocytes"[MeSH Terms] OR “oocytes"[All Fields] OR “oocyte"[All Fields] OR “oocytic"[All Fields]) AND “pick-up"[All Fields])
#2	"vaginal douching"[MeSH Terms] OR (“vaginal"[All Fields] AND “douching"[All Fields]) OR “vaginal douching"[All Fields] OR (“vaginal douching"[MeSH Terms] OR (“vaginal"[All Fields] AND “douching"[All Fields]) OR “vaginal douching"[All Fields] OR (“douching"[All Fields] AND “vaginal"[All Fields]) OR “douching vaginal"[All Fields]) OR (“vaginal douching"[MeSH Terms] OR (“vaginal"[All Fields] AND “douching"[All Fields]) OR “vaginal douching"[All Fields] OR (“vaginal"[All Fields] AND “lavage"[All Fields]) OR “vaginal lavage"[All Fields]) OR (“vaginal douching"[MeSH Terms] OR (“vaginal"[All Fields] AND “douching"[All Fields]) OR “vaginal douching"[All Fields] OR (“lavage"[All Fields] AND “vaginal"[All Fields]) OR “lavage vaginal"[All Fields]) OR (“vaginal douching"[MeSH Terms] OR (“vaginal"[All Fields] AND “douching"[All Fields]) OR “vaginal douching"[All Fields] OR (“vaginal"[All Fields] AND “irrigation"[All Fields]) OR “vaginal irrigation"[All Fields]) OR (“vaginal douching"[MeSH Terms] OR (“vaginal"[All Fields] AND “douching"[All Fields]) OR “vaginal douching"[All Fields] OR (“irrigation"[All Fields] AND “vaginal"[All Fields])) OR ((“vagina"[MeSH Terms] OR “vagina"[All Fields] OR “vaginal"[All Fields] OR “vaginally"[All Fields] OR “vaginals"[All Fields] OR “vaginitis"[MeSH Terms] OR “vaginitis"[All Fields] OR “vaginitides"[All Fields]) AND (“washed"[All Fields] OR “washes"[All Fields] OR “washing"[All Fields] OR “washings"[All Fields])) OR ((“vagina"[MeSH Terms] OR “vagina"[All Fields] OR “vaginal"[All Fields] OR “vaginally"[All Fields] OR “vaginals"[All Fields] OR “vaginitis"[MeSH Terms] OR “vaginitis"[All Fields] OR “vaginitides"[All Fields]) AND (“preparate"[All Fields] OR “preparates"[All Fields] OR “preparation"[All Fields] OR “preparations"[All Fields] OR “preparative"[All Fields] OR “preparatively"[All Fields] OR “prepare"[All Fields] OR “prepared"[All Fields] OR “prepares"[All Fields] OR “preparing"[All Fields]))
	((#1) AND (#2))

### Inclusion criteria

(1) Experimental group with iodine used to wash the vagina as an intervention for patients need vaginal prep before oocyte retrieval. (2) Control group with other vaginal douching agents used to wash the vagina before oocyte retrieval. (3) Human clinical trials. (4) Outcome indicators including at least one of the following: OPU-PI and IVF outcome (including number of oocytes, fertilization rate, clinical pregnancy rate).

### Exclusion criteria

(1) Studies with incomplete or unreported data (2) Studies from non-trials [including systematic reviews, animal studies, protocols, conference abstracts, case reports or meta-analysis.

### Study selection

The literature was screened and excluded using the literature management soft-ware Endnote X9. Two researchers first screened the titles of the literature for duplication, non-trial studies, review papers, conference papers, protocols and meta-analysis. The abstracts of the literature were then read by two researchers to identify literature for inclusion and to exclude literature. Finally, the remaining literature was read in full by both researchers and further identified for inclusion. During this process, both researchers independently screened the literature and finally compared the remaining literature, if it was the same then it was finally included, if it was different then it was discussed and resolved by a third researcher.

### Data extraction

A eight-item, standardized and pre-selected data extraction form was used to record data for inclusion in the study under the following headings: (1) author, (2) year of publication, (3) country, (4) population, (5) sample size, (6) details of the intervention, and (7) outcome (Table 2).

**Table 2:** Characteristics of the studies included in the meta-analysis.

Author	Country	Year	Population	Total	Intervention	Control	Outcome
YUAN Yan	China	2019	Patients need vaginal prep before oocyte retrieval in IVF-ET	A: 20 B: 20 C: 20	Using Iodine and Ozone to wash the vagina before oocyte retrieval	Using Potassium Permanganate to wash the vagina	OPU-PI
YOU Yi-qiu	China	2015	Patients need vaginal prep before oocyte retrieval in IVF-ET	A: 30 B: 30 C: 30	Using Iodine and Ozone to wash the vagina before oocyte retrieval	Using Potassium Permanganate to wash the vagina	OPU-PI
WU Yuan-fei	China	2014	Patients need vaginal prep before oocyte retrieval in IVF-ET	T:2244 C:1265	Using Chlorhexidine acetate + Saline to wash the vagina before oocyte retrieval	Using Sterile saline only to wash the vagina	OPU-PI FR CP
TAN Chang-xiu	China	2014	Patients need vaginal prep before oocyte retrieval in IVF-ET	T:3232 C:2350	Using Iodine + Saline to wash the vagina before oocyte retrieval	Using Sterile saline only to wash the vagina	OPU-PI FR CP
Funabiki	Japan	2014	Patients need vaginal prep before oocyte retrieval in IVF-ET	T:1216 C:956	Using Iodine + Saline to wash the vagina before oocyte retrieval	Using Sterile saline only to wash the vagina	OPU-PI
Hannoun	American	2008	Patients need vaginal prep before oocyte retrieval in IVF-ET	T:356 C:356	Using Iodine + Saline to wash the vagina before oocyte retrieval	Using Iodine only to wash the vagina	OPU-PI CP
Tsai	TAIWAN in China	2005	Patients need vaginal prep before oocyte retrieval in IVF-ET	T:52 C:56	Using Iodine + Saline to wash the vagina before oocyte retrieval	Using Sterile saline only to wash the vagina	OPU-PI FR CP
Van	Netherlands	1992	Patients need vaginal prep before oocyte retrieval in IVF-ET	T:160 C:174	Using Iodine to wash the vagina before oocyte retrieval	Using Sterile saline only to wash the vagina	OPU-PI FR CP

OPU-PI, oocyte pick-up related pelvic infection; FR, fertilization rate; CP, clinical pregnancy rate; T, experimental group; C, control group.

### Risk of bias of studies

Two researchers independently assessed the risk of bias (ROB), in accordance with the Cochrane Handbook version 5.1.0 tool for assessing ROB in RCTs. The following domains were included: (1) randomized sequence generation, (2) treatment allocation concealment, blinding of (3) participants and (4) personnel, (5) incomplete outcome data, (6) selective reporting and (7) other sources of bias. Trials were categorized into three levels of ROB: high risk (five or more components of high ROB potentially existed), moderate risk (three or four components of high ROB potentially existed) and low risk (two or less components of high ROB potentially existed) (12).

### Data analysis

We used Stata software (version 15.1) and performed NMA aggregation and analysis using Markov chain Monte Carlo simulation chains in a Bayesian-based framework according to the PRISMA NMA instruction manual (13). We will use the nodal method to quantify and demonstrate the agreement between indirect and direct comparisons, calculated through the instructions in the Stata software, and if the *P*-value > 0.05. the consistency test passes (14).

Stata software is used to present and describe network diagrams of different movement interventions. In the generated network diagrams, each node represents a different motor intervention and a different control condition, and the lines connecting the nodes represent direct head-to-head comparisons between interventions. The size of each node and the width of the connecting lines are proportional to the number of studies (15). All variables in this study are categorical variables and are expressed as odds ratios (OR) with 95% confidence intervals (CI) and analysis. As there are certainly potential differences across studies, we chose a random effects model for analysis rather than a fixed effects model (16).

## Results

### Study and identification and selection

**Figure 1:**
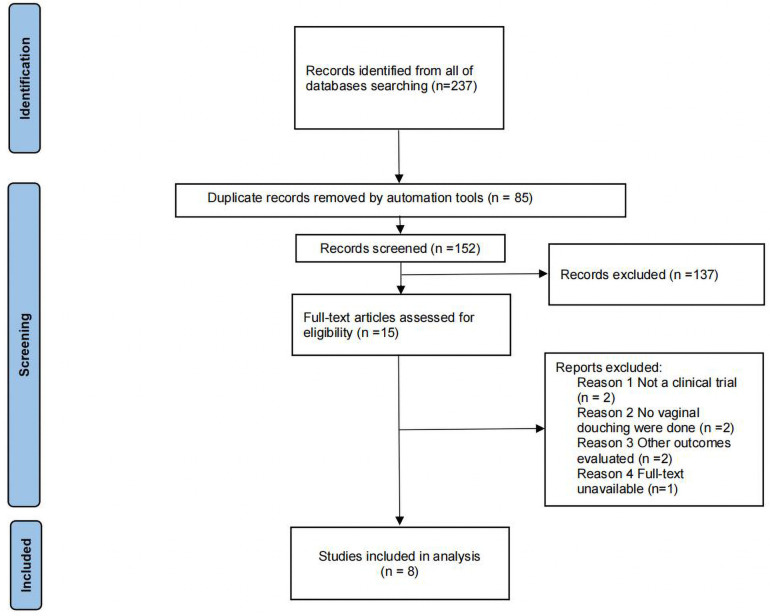
Flow diagram of literature selection.

A total of 231 documents were retrieved from the electronic database, and an additional 6 documents were manually searched. After eliminating duplicates, the remaining 152 documents were read for titles and abstracts, and 137 documents were again excluded. The remaining 15 documents were read in full and 7 documents were again excluded (for reasons including: not a clinical trial, no vaginal douching were done, other outcomes evaluated and full-text unavailable), leaving a final remaining 8 documents to be included in this study (Figure 1).

### Quality assessment of the included studies

**Figure 2:**
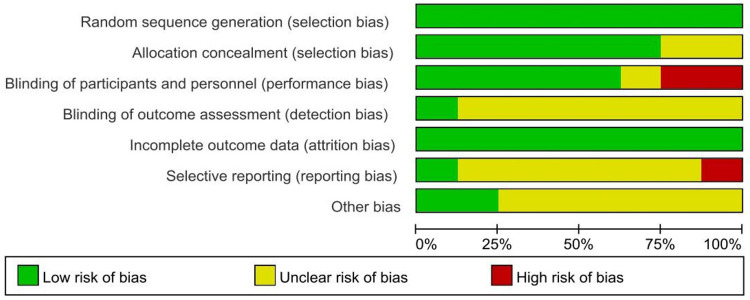
Risk of bias graph.

**Figure 3:**
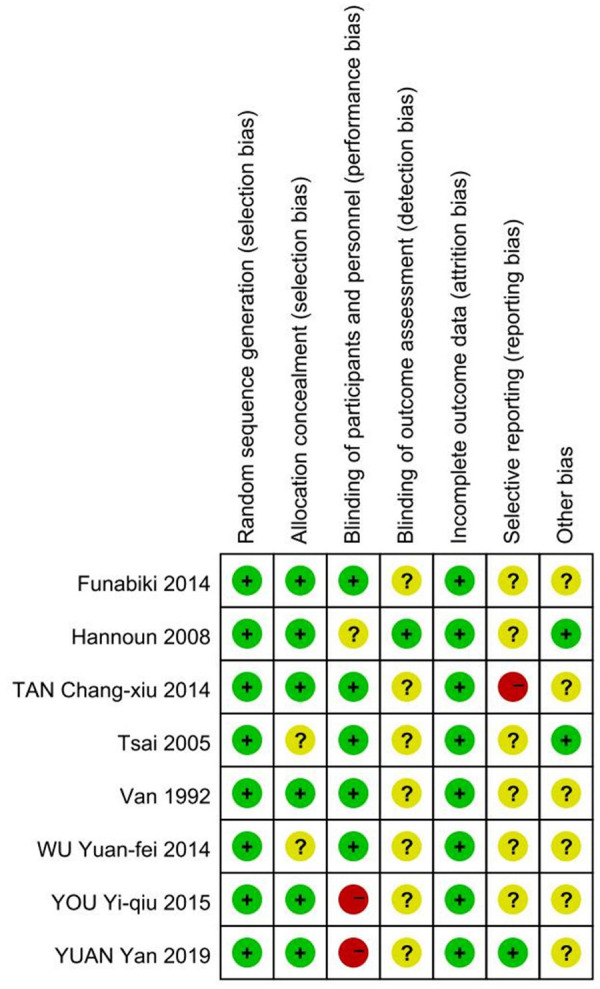
Risk of bias summary.

All of the included studies were defined as low risk and simultaneously achieved avoiding selection bias and attrition bias, but as the intervention in these studies was vaginal douching, it was difficult to achieve simultaneous blinding of subjects and measurers as both the patients themselves and their relatives had to sign an informed consent form before the experiment was conducted. Specific details will be presented in Figures 2, 3.

### Characteristics of the included studies

In total, we included studies from 8 clinical trials, which included 12,567 patients need vaginal prep before oocyte retrieval in IVF-ET. The vaginal douching agents in intervention groups included Ozone (2 studies) (17, 18), Iodine only (3 studies) (17–19), Iodine followed by saline(4 studies) (20–23) and Chlorhexidine acetate followed by saline (24). Interventions in the control group included using Potassium Permanganate (2 studies) (17, 18), sterile saline (5 studies) (19–21, 23, 24), iodine (1 study) (22) to wash the vagina before OPU. All of the studies reported OPU-PI as an outcome indicator, 5 studies reported clinical pregnancy rate as an outcome indicator and 4 studies reported fertilization rate as an outcome indicator. There were 5 studies from China and other 3 studies were from America, Japan and Netherlands, respectively. The characteristics of the included studies are shown in Table 2.

### Network meta-analysis

**Figure 4:**
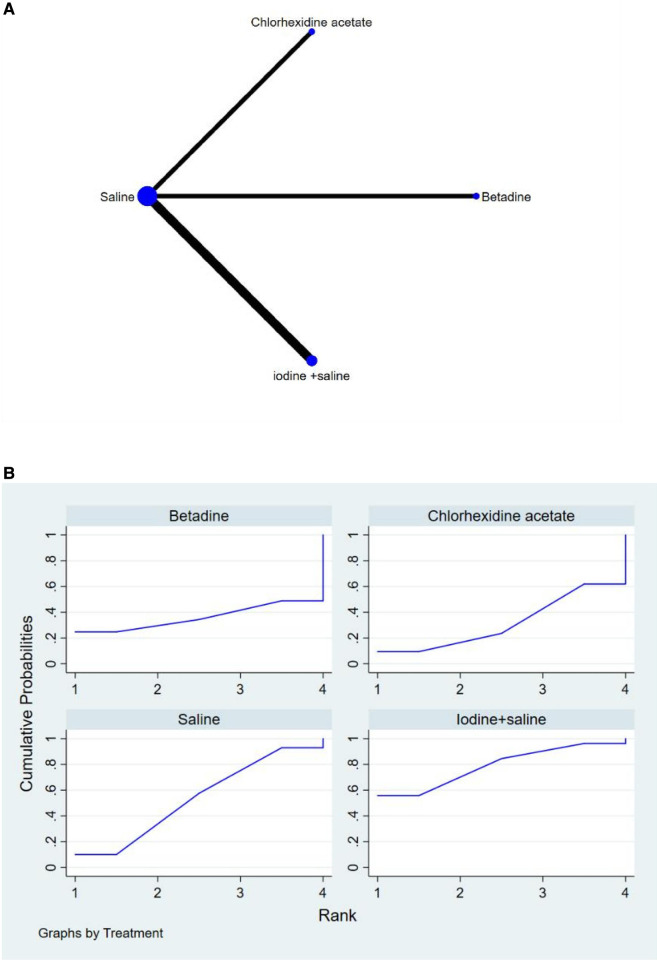
(**A**) NMA figure for FR (**B**) SUCRA plot for FR.

**Figure 5:**
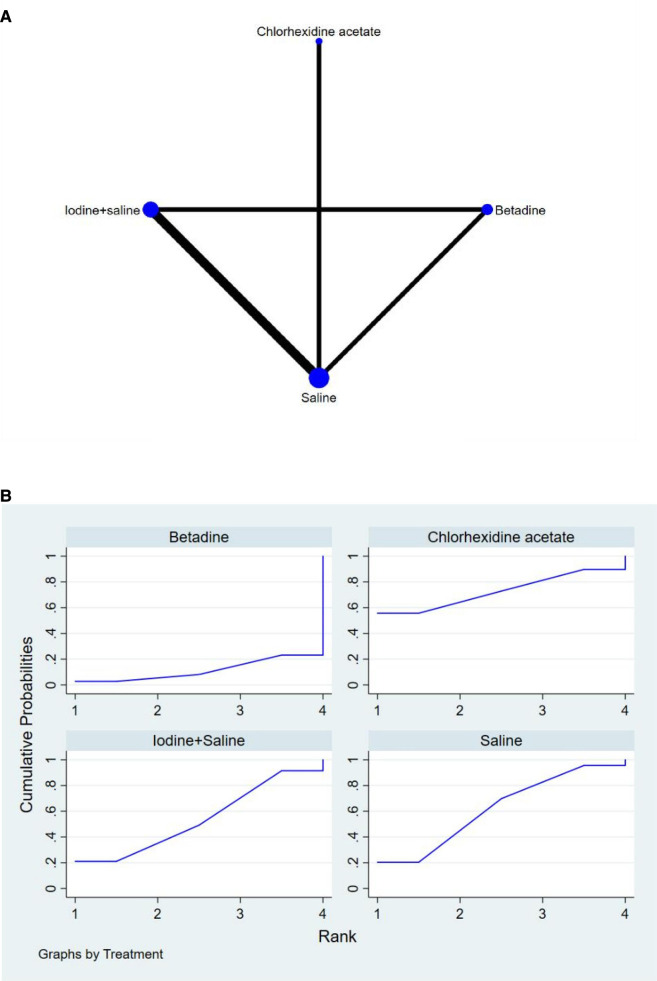
(**A**) NMA figure for CP (**B**) SUCRA plot for CP.

**Figure 6:**
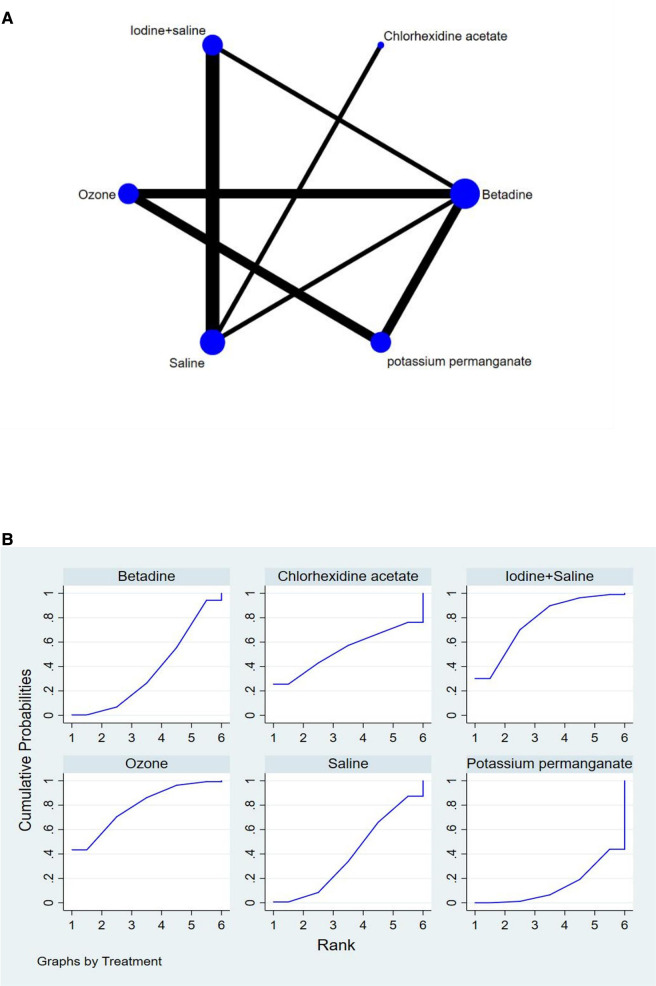
(**A**) NMA figure for OPU-PI (**B**) SUCRA plot for OPU-PI.

The full NMA figure will be shown in Figures 4A, 5A, 6A.

#### Fertilization rate (Fr)

All *P*-values for indirect and direct comparisons between all studies were tested for consistency and inconsistency, and all *P*-values were greater than 0.05, indicating that the effect of consistency between studies was acceptable.

The results of the Network meta-analysis showed that relative to the 4 different vaginal douching agents, Betadine, Chlorhexidine acetate followed by saline, Saline, Iodine followed by saline. The probability ranking of the different vaginal douching agents in terms of improving fertilization rate was ranked first in the SUCRA for Iodine followed by saline (SUCRA was shown in Figure 4B). A comparison between the two different interventions will be shown in Table 3.

**Table 3:** League table on FR.

Iodine + saline	Saline	Betadine	Chlorhexidine acetate
Iodine + saline	0.95 (0.83,1.08)	0.87 (0.55,1.36)	0.89 (0.71,1.12)
**1.06 (0.93,1.21)**	Saline	0.92 (0.60,1.41)	0.94 (0.78,1.13)
**1.15 (0.73,1.80)**	**1.09 (0.71,1.67)**	Betadine	1.02 (0.64,1.63)
**1.12 (0.90,1.41)**	**1.06 (0.88,1.28)**	**0.98 (0.61,1.56)**	Chlorhexidine acetate

#### Clinical pregnancy rate (Cp)

All *P*-values for indirect and direct comparisons between all studies were tested for consistency and inconsistency, and all *p*-values were greater than 0.05, indicating that the effect of consistency between studies was acceptable.

The results of the network meta-analysis showed that relative to the 4 different vaginal douching agents, Betadine, Chlorhexidine acetate followed by saline, Saline, Iodine followed by saline. The probability ranking of the different vaginal douching agents in terms of improving clinical pregnancy rate was ranked first in the SUCRA for Chlorhexidine acetate followed by saline (SUCRA was shown in Figure 5B). A comparison between the two different interventions will be shown in Table 4.

**Table 4:** League table on CP.

Chlorhexidine acetate	Saline	Iodine + saline	Betadine
Chlorhexidine acetate	0.85 (0.36,2.01)	0.79 (0.28,2.26)	0.51 (0.16,1.60)
1.17 (0.50,2.76)	Saline	0.92 (0.50,1.70)	0.60 (0.28,1.27)
1.27 (0.44,3.64)	1.08 (0.59,2.00)	Iodine + saline	0.65 (0.31,1.35)
1.96 (0.62,6.15)	1.67 (0.78,3.57)	1.54 (0.74,3.22)	Betadine

#### Ovum pick-up related pelvic infection rate (OPU-Pi)

All *P*-values for indirect and direct comparisons between all studies were tested for consistency and inconsistency, and all *p*-values were greater than 0.05, indicating that the effect of consistency between studies was acceptable.

The results of the network meta-analysis showed that relative to the 6 different vaginal douching agents, Betadine, Chlorhexidine acetate followed by saline, Saline, Iodine followed by saline, Ozone [MD = 0.09, 95% CI =(0.01, 0.76)], Potassium permanganate. The probability ranking of the different vaginal douching agents in terms of reducing OPU-PI rate was ranked first in the SUCRA for Ozone (SUCRA was shown in Figure 6B). A comparison between the two different interventions will be shown in Table 5.

**Table 5:** League table on OPU-PI.

Ozone	Iodine + saline	Chlorhexidine acetate	Saline	Betadine	Potassium permanganate
Ozone	1.20 (0.05,30.88)	2.48 (0.02,353.42)	4.40 (0.21,91.64)	5.45 (0.61,48.40)	10.76 (1.31,88.48)
0.83 (0.03,21.51)	Iodine + saline	2.07 (0.03,150.16)	3.68 (0.65,20.62)	4.54 (0.41,50.35)	8.98 (0.62,130.34)
0.40 (0.00,57.32)	0.48 (0.01,34.97)	Chlorhexidine acetate	1.77 (0.04,89.43)	2.19 (0.03,187.93)	4.33 (0.04,432.06)
0.23 (0.01,4.72)	0.27 (0.05,1.53)	0.56 (0.01,28.43)	Saline	1.24 (0.15,10.17)	2.44 (0.22,27.23)
0.18 (0.02,1.63)	0.22 (0.02,2.44)	0.46 (0.01,39.08)	0.81 (0.10,6.65)	Betadine	1.98 (0.61,6.38)
**0.09** **(0.01,0.76)**	0.11 (0.01,1.62)	0.23 (0.00,23.03)	0.41 (0.04,4.56)	0.51 (0.16,1.63)	Potassium permanganate

### Publication bias test

**Figure 7:**
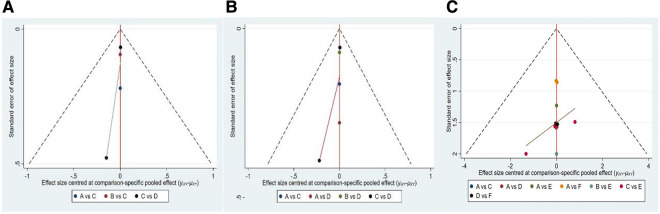
Funnel plot on publication bias (**A**) FR (**B**) CP (**C**) OPU-PI.

We constructed separate funnel plots for all outcome indicators to test for possible publication bias. Visual inspection of the funnel plots did not reveal any significant publication bias (25). Details as shown in Figure 7.

## Discussion

In this study we compared different vaginal douching agents to decrease OPU-PI and improve IVF outcome in people who need vaginal prep before oocyte retrieval. A total of 8 studies including 6 different vaginal preparation protocols were included, including 12,567 patients, which is a fairly large sample size. Our study showed that using iodine followed by sterile saline to wash vagina before OPU was the best intervention in terms of increasing fertilization rate, Chlorhexidine acetate followed by saline was the best solution in terms of increasing clinical pregnancy rate, but using Ozone to wash vagina showed better results in terms of decreasing the OPU-PI. Overall, however, we believe that there are three appropriate vaginal douching agents for ensuring the security of OPU while improving IVF outcome in patients who need IVF treatment.

As an important part of fertility treatment, OPU is generally viewed as a low-risk of procedure. Symptomatic pelvic infection following transvaginal oocyte retrieval is a rare, but serious complication (26). Cleansing of the vagina/cervix is therefore widely believed should be done prior to OPU to minimize bacterial vaginal/cervical contamination (27). Many kinds of disinfection protocols before OPU were reported but there is no evidence on the safety of those antiseptic methods, and how different vaginal douching agents may influence reproductive outcomes and post-OPU complications (28). Our results demonstrate that ozone has a statistically significant beneficial effect on the decreasing OPU-PI compared to other agents, which is consistent with the results of other animal studies (29, 30). As a highly reactive molecule, ozone is emerging as a new antifungal agent for vaginal/pelvic infection in recent years (31). One of the common causes of OPU-PI is Candida albicans, a study tested that ozone was highly effective on the yeast form of Candida albicans and it can inhibit the formation of germ tubes in Candida albicans (32). Besides Candida albicans, other clump of bacteria such as Staphylococcus aureus, Escherichia coli, and M. urealyticum that may cause the acute pelvic inflammatory disease were also confirmed sensitive to ozone (33). In addition, Merhi Z et al. found that ozone therapy could have beneficial effect on tubal occlusion, protect patients from endometritis and vaginitis, and might also protect ovaries from ischemia and oocyte loss and finally might lead to less formation of pelvic adhesions (34). Taken together, the key findings of the study present evidence that ozone may aid in decreasing the vagina flora and protect the fertility preservation of infertility patients.

In addition, fertilization rate seems to be a significantly important part of IVF outcome. Some disinfection agents previously confirmed that might act on the oocyte's cell membrane (35) and might have also been associated with epithelial toxicity (36), which can not be safe for oocytes (37), and the quality of oocytes were significant positive predictors of fertilization rate (38). Also, If there were inadequate numbers of mature oocytes are available after OPU, fertilization failure (TFF) occurs and can reoccur in subsequent cycles (39). If povidone–iodine preparations were used before OPU, the remained iodine could contaminate the aspirates and in that way damage the oocytes, or they might be imbibed by the cervical plug and thus gain access to the uterine cavity (40). Another possibility is that traces of Betadine which remain in the cervical mucus plug may be introduced into the uterine cavity with embryo transfer (41). In our study, it was shown that the fertilization rate are lower when betadine solution which is not rinsed with saline solution are employed. Iodine followed by thoroughly saline douching was the most effective agents on improving fertilization rate among the different agents included in this study. The results are the same as the original study (42). Even though the vaginal anatomy is such that traces of Iodine can remain, the results of this study attributed to the fact that all the antiseptic solution should be completely flushed away before oocyte retrieval.

The increase in the clinical pregnancy rate is difficult to explain. The increase in the clinical pregnancy rate has been attributed to the low risk of pregnancy loss rate, controlled ovarian hyperstimulation (COH) protocol, cycle type and serum hCG level 14 days after transfer (43, 44). In addition, even the unicornuate uterus could affects the clinical pregnancy rate in IVF-ET (45). Therefore, the high clinical pregnancy rate not only depends on the vaginal preparations before OPU. At the same time, we got the conclude that Chlorhexidine acetate followed by saline washing may be the most beneficial agents in improving the clinical pregnancy.

Overall our study has some clinical implications, firstly Ozone and Iodine followed by saline have a significant effect in decreasing OPU-PI without compromising the outcome of IVF treatment, and furthermore doctors can make a decision on which vaginal douching agents to use before OPU based on the data of this study.

## Strengths and limitations

Firstly our study included 8 studies and 12,567 patients, which is a very large sample size, and we also built on the original review on the vaginal douching agents for people who need oocyte retrieval in IVF-ET by including saline, Iodine, Iodine followed by saline, Ozone, Potassium permanganate and Chlorhexidine acetate followed by saline, which provides newer and more comprehensive evidence-based recommendations.

Secondly, our study shares some limitations with the studies on which it is based. Although we made every effort to control for study heterogeneity when including these original studies, heterogeneity between studies was unavoidable (e.g., the proportion of studies by region and between clinical characteristics).

Finally, in our study, readers should interpret the results with caution because of the small number of studies and the limited head-to-head direct comparative evidence for some interventions. It also highlights the need for further expansion of relevant studies.

## Conclusions

In our study, Ozone is the most recommended antiseptic solution for patients who want to decrease infection rate after OPU; Iodine followed by thoroughly saline douching is the most recommended agents for patients who want to ensuring the quality of oocytes.

Overall, however, we recommended using Iodine followed by thoroughly saline washing as the antiseptic solutions for clinicians in order to decreasing OPU-PI after OPU without affecting IVF outcome.

## Data Availability

The original contributions presented in the study are included in the article/Supplementary Material, further inquiries can be directed to the corresponding author/s.
